# Safety and Tolerability of Both Arm Ischemic Conditioning in Patients With Major Depression: A Proof of Concept Study

**DOI:** 10.3389/fpsyt.2020.00570

**Published:** 2020-06-18

**Authors:** Zuowei Wang, Xujuan Li, Ningning Li, Leping Huang, Jiawen Liu, Bixiu Yang, Jingquan Shi, Yue Fei, Xunming Ji, Keming Gao, Ming Ren

**Affiliations:** ^1^ Division of Mood Disorders, Hongkou District Mental Health Center, Shanghai, China; ^2^ School of Medicine, Shanghai University, Shanghai, China; ^3^ Department of Psychology, Naval Medical University, Shanghai, China; ^4^ Department of Psychiatry, Shulan (Hangzhou) Hospital, Affiliated to Zhejiang Shuren University Shulan International Medical College, Hangzhou, China; ^5^ Department of Psychology, Wuxi Mental Health Center, Wuxi, China; ^6^ Department of Neurosurgery, Xuanwu Hospital, Capital Medical University, Beijing, China; ^7^ Mood and Anxiety Clinic in the Mood Disorders Program of the Department of Psychiatry, University Hospital Cleveland Medical Center, Cleveland, OH, United States; ^8^ Department of Psychiatry, Case Western Reserve University School of Medicine, Cleveland, OH, United States; ^9^ Department of Neurology, Xuanwu Hospital, Capital Medical University, Beijing, China

**Keywords:** major depressive disorder, remote ischemic preconditioning, safety, tolerability, effectiveness

## Abstract

**Purpose:**

A substantial proportion of patients with major depressive disorder (MDD) does not respond or cannot tolerate to currently available treatments. This study was to assess the safety and tolerability of Remote Limb Ischemic Preconditioning (RLIPC) as an adjunctive therapy in patients with MDD.

**Patients and Methods:**

Enrolled patients underwent RLIPC, five cycles of simultaneous bilateral arm ischemia, 5 min and followed by reperfusion of each cycle, and once daily for eight consecutive weeks. Depression and anxiety severity, and quality of life were assessed every 2 weeks. Descriptive analysis was used for safety and tolerability data.

**Results:**

Thirty-seven participants completed at least one RLIPC. Twenty-four of them (64.9%) completed the study. Twelve patients prematurely discontinued the study due to poor adherence, and one due to a mild side effect. The changes in HRSD-17, GAD-7 and QOL-6 total scores from baseline to the endpoint were significant from the end of second week treatment onwards. The responder and remission rates were 59.46% (22/37) and 54.05% (20/37) at the endpoint, respectively.

**Conclusion:**

RLIPC was safe and well tolerated, and may be effective in reducing depressive symptoms in patients with MDD. Large studies are warranted to test its efficacy and safety as monotherapy or adjunctive therapy in the treatment of MDD.

## Introduction

Major depressive disorder (MDD) is one of the most common mental disorders in the general population and a major public health problem across the world. It is associated with high rates of morbidity and mortality (1). According to the World Health Organization, MDD affects approximately 4.4% of the world’s population, has high occupational and economic impact, and will become the second leading globe burden of disease by 2020 (2). In China, the latest national epidemiological survey found that the 12-month and life time prevalence of depressive disorders was 3.6 and 6.8%, and MDD was 2.1 and 3.4% respectively (3).

Although a number of effective treatments are available, a substantial proportion of patients with MDD does not respond or cannot tolerated to the first-line antidepressants (4). In the Sequenced Treatment Alternatives to Relieve Depression (STAR*D) trials, only about one-half of patients responded, and one-third of remitted with the first level of antidepressant treatments (5, 6). After failing an antidepressant for a current depressive episode, switching to a different antidepressant in the same class or a different class, or augmenting with an antidepressant, mood stabilizer, or antipsychotics is a common practice. Non-pharmacological augmentation such as repetitive transcranial magnetic stimulation (rTMS) has become available to manage inadequate response to the initial treatment of an antidepressant(s) (7–9). Although, rTMS does not have severe side effect, a 5 day per week schedule for a minimal 4–6 weeks treatment to determine its benefit is cumbersome for those who have already struggled socially and financially. More convenient and inexpensive approaches with fewer short- and long-term side effect are worthy of exploration. Remote ischemic preconditioning (RIPC) appears to fit this profile.

RIPC consists of several cycles of brief and nonlethal limb ischemia (10, 11). With a series of transient ischemic episodes, it can activate multiple endogenous protective mechanisms to attenuate ischemic injury and alleviate dysfunction to distant organs due to acute reduction of blood supply (12). RIPC may also have neuroprotective effects through a variety of potential mechanisms, including regulation of specific neural pathway and signal transduction, inhibition of inflammatory and immune responses, decrease of oxidative stress and calcium overload, attenuation of glutamate excitotoxicity, and suppression of apoptosis (10–14). Its neuroprotective effect was observed in participants with symptomatic intracranial arterial stenosis (ICAS) and carotid artery stenosis (CAS), which could reduce the new brain lesions and the impairment of neural circuits and connectivity, and decrease the risk of various psychiatric or neurological conditions (e.g., cognitive decline, dementia and depression) (15–17). A recent study found that RIPC could prevent the progression of white matter hyperintensities and ameliorate headache, dizziness, sleeping disorder and cognitive impairment in elderly patients (83.5 ± 2.3 years) who had intracranial atherosclerotic stenosis (18).

Although the neurobiology of MDD remains unknown, the involvement of neurotrophic factors, inflammatory cytokines, the hypothalamus–pituitary–adrenal axis, and glutamate receptors in the pathophysiology of depression have been reported (19, 20), which was overlapped and consistent with the potential mechanisms of RIPC. RIPC has effect on a variety of changes in peripheral and central nervous system, which may reverse the neurobiological changes and improve depressive or related symptoms (e.g., cognition, sleep, and physical symptoms) (16, 18). Therefore, we conducted a proof of concept study to assess the safety and tolerability of Remote Limb Ischemic Preconditioning (RLIPC) as an adjunctive therapy in patients with MDD who were treated with an antidepressant for a current depressive episode.

## Materials and Methods

### Subjects

This study was a single-arm, open-label, prospective, multicenter clinical trial. The study protocol was approved by the Institutional Review Board of Hongkou District Mental Health Center (Shanghai, China). Written informed consents were obtained from each participant before any study-related procedure was performed.

All patients were inpatients from Hongkou District Mental Health Center of Shanghai, Shulan (Hangzhou) Hospital of Zhejiang Province, and Wuxi Mental Health Center of Jiangsu Province. The study was conducted from the January of 2018 to the June of 2019. Patients who met the following criteria were enrolled into the study: 1) age of 18–65 years old; 2) diagnosed with MDD according to the fifth edition of the Diagnostic and Statistical Manual of Mental Disorders (DSM-5); 3) a score of ≥14 on the 17- item Hamilton Rating Scale for Depression (HRSD-17) at the screening visit; 4) had the capacity for the informed consent for the study.

Patients with a history of manic or hypo-manic episode were excluded from this study. Patients were also excluded if they had any of the following physical diseases: 1) Extracranial and intracranial arterial stenosis verified with structural MRI, and other intracranial abnormalities such as infection, tumor and bleeding; 2) Known bleeding disorders or platelet counts <100 * 10^9^/L, history of retinal and visceral hemorrhage, or on thrombolytic therapy; 3) Refractory hypertension defined as systolic pressure >180 mmHg or diastolic pressure >110 mmHg with evidence-based treatments; 4) Severe renal failure define as the rate of creatinine clearance <0.6 ml/s or serum creatinine >265 umol/l; 5) severe hepatic failure defined as serum alanine aminotransferase (ALT) or aspartate aminotransferase (AST) three times higher than the upper normal limit; 6) History of heart attack or a diagnosis of coronary artery disease; 7) Peripheral vascular disease of the upper extremity such as severe subclavian artery stenosis verified with MRI; or 8) Soft damage, vascular injury or fracture in the upper extremities. In addition, female patients who were pregnant, planning to be pregnant, or breast feeding during the study period were also excluded.

### Antidepressant Treatment

For patients who were taking an antidepressant(s) for two weeks or more and met the inclusion criteria without any exclusion criteria, the RLIPC was added to the ongoing treatment regimen. For patients who did not take any medication or took an antidepressant(s) for less than two weeks for their depression at screening, an antidepressant was initiated and titrated up to a therapeutic dose within 2 weeks. For those who tolerated with the newly initiated antidepressant and continued meeting the inclusion criteria, the RLIPC was added. The initiation of any other psychotropic medication including typical/atypical antipsychotic agents, mood stabilizers, anticonvulsants, and stimulants, psychotherapy and physical treatment (rTMS and ECT) were not permitted during the study period. If necessary, short half-life hypnotics of benzodiazepines or non-benzodiazepines could be prescribed to manage anxiety, agitation and insomnia.

### Implementation of RLIPC

The RLIPC application consisted of five cycles of simultaneous bilateral upper arm ischemia through a complete blocking of the arterial and venous blood flow to the forearms. Each cycle lasted for 5 min and followed by reperfusion for 5 min (16). An electric autocontrol device with cuffs was used to produce a pressure of 200 mmHg for ischemic response. The device Ischemic Precondition Training Instrument IPC-906 was provided by Beijing Renqiao Cardio-cerebrovascular Disease Prevention Research Nantong Co., Ltd, China. The RLIPC process could be stopped at any time if the patient experienced any intolerable discomfort or side effect. The enrolled patients underwent RLIPC once daily for eight consecutive weeks under the supervision and guidance of nurses and research assistants.

### Safety Monitoring

Treatment-emergent adverse event was assessed and recorded at every treatment, including inability to tolerate RLIPC that leads to the discontinuation from the study and objective signs of tissue or neurovascular injury resulting from RLIPC procedure (16). The severity of an adverse event was determined by a research staff member. The forms of adverse event (AE) and serious adverse event (SAE) reporting were completed in according to the requirement from Institutional Review Board of Hongkou District Mental Health Center (Shanghai, China). Clinical laboratory tests including complete blood count, electrolytes, liver and kidney function tests, urine pregnancy test (if applicable), and ECG examination were performed at baseline and at the end of study.

### Efficacy Assessment

The HRSD-17 was administered by the trained psychiatrists and psychologists. The Generalized Anxiety Disorder Scales 7-item (GAD-7) and quality of life 6-item (QOL-6) were completed by the patients. All these assessments were performed at baseline, week 2, week 4, week 6, and week 8.

### Statistical Analysis

Demographic characteristics, and the rate of AE, SAE, response and remission were analyzed with descriptive statistics. The safety population included all patients who received at least one RLIPC treatment. The changes from baseline to endpoint in the total scores of HRSD-17, GAD-7 and QOL-6 were analyzed with ANOVA for repeatedly measured data based on the intent-to-treat sample. Paired t-test was also used to analyze the difference in changes in HRSD-17, GAD-7 and QOL-6 between baseline and following up visits. Depression response and remission were defined as a 50% decrease in the HRSD-17 total score from baseline to the endpoint and a HRSD-17 total score ≤7 at the endpoint, respectively. The last observation carried forward (LOCF) strategy was used to impute missing values of efficacy assessment for those subjects who did not complete the study. All statistical analyses were carried out by using SPSS 25.0 software program. Criterion for statistical significance was set at α = 0.05.

## Results

### Demographics and Baseline Clinical Characteristics

Forty patients who met the criteria of inclusion and did not meet any criterion of exclusion were included in the study after a screening assessment. Thirty-seven participants who completed at least one RLIPC procedure were included for the analysis. These patients were more likely to be female (n = 30, 81.1%), married (n = 27, 73.0%), and employed (n = 17, 45.9%) (**Table 1**). The education, age, and baseline severity scores of HRSD-17, GAD-7 and QOL-6 at study entry were also shown in **Table 1**. All patients were Han Chinese.

**Table 1:** Demographics and clinical characteristics of enrolled patients with depression.

Characteristic	N	%
*Race (Han)*	37	100
*Gender* Male Female	7 30	18.9 81.1
*Marriage* Unmarried Married Divorced and Widowed	7 27 3	18.9 73.0 8.1
*Occupation* Employed Retired Unemployed Student	17 11 3 6	45.9 29.7 8.1 16.2
	*Mean*	*SD*
*Education (years)*	11.51	4.58
*Age (years)*	42.54	15.98
*Symptom severity at baseline* HRSD-17 GAD-7 QOL-6	20.51 10.11 15.00	8.50 4.94 3.33

HRSD-17, 17-item Hamilton Rating Scale for Depression; GAD-7, Generalized Anxiety Disorder Scales 7-item; QOL-6, Quality of Life 6-item.

### Disposition

During the 8-week study period, 64.9% (24/37) patients completed the study and twelve patients prematurely discontinued the study due to poor adherence defined as continuous interruption for more than two days in a row at any time (n = 5) or to be discharged (n = 7). Among those patients who prematurely discontinued, four patients discontinued at the second weekend, seven patients at the fourth weekend and, one patient at the eighth weekend, respectively.

### Adverse Events

Of 37 patients enrolled in the study, only one patient had a mild side effect with numbness of limbs and petechiae of skin under the pressure cuff area during the first two weeks of RLIPC. The numbness of limbs and petechiae of skin disappeared one week after stopping the RLIPC, and the patient discontinued the study.

### Efficacy Data

**Figure 1:**
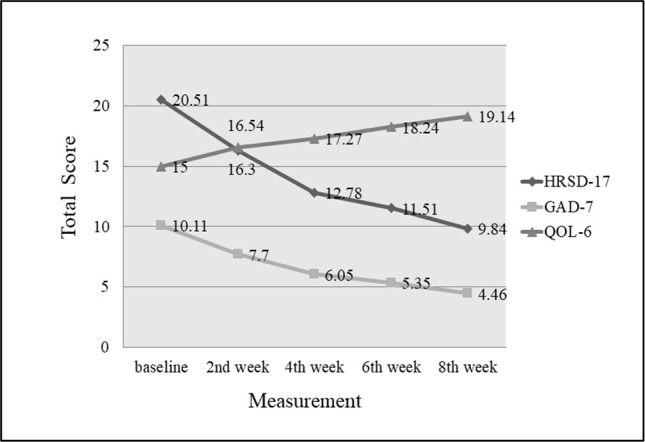
Changes of HRSD-17, GAD-7 and QOL-6 total score from baseline to endpoint. Compared with baseline, total scores of HRSD-17 and GAD-7 decreased significantly, and QOL-6 increased significantly by the end of second week (*P < *0.001).

The decrease in HRSD-17 and GAD-7 total scores from baseline to the endpoint were significant, 20.51 ± 8.50 vs. 9.84 ± 8.00 (*P <* 0.001) and 10.11 ± 4.94 vs. 4.46 ± 3.33 (*P <*0.001), respectively (**Table 2**). The increase in total scores of QOL-6 from baseline and the endpoint were also significant, 15.00 ± 3.33 vs. 19.14 ± 2.78 (*P <*0.001) (**Table 2**). The significant changes in the total scores of HRSD-17, GAD-7 and QOL-6 occurred from the end of second week treatment onwards (**Figure 1**). The responder and remission rates were 59.46% (22/37, 95% CI as 43.40–74.25%) and 54.05% (20/37, 95% CI as 38.13–69.43%), respectively.

**Table 2:** Efficacy outcomes of adjunctive remote limb ischemic preconditioning to antidepressants in patients with depression (N = 37).

Measure	Mean	SD	F	P
*HRSD-17* Baseline Endpoint^a^ Change	20.51 9.84 −10.68	8.50 8.00 6.84	21.08	<0.001
*GAD-7* Baseline Endpoint^a^ Change	10.11 4.46 −5.65	4.94 3.33 4.72	12.83	<0.001
*QOL-6* Baseline Endpoint^a^ Change	15.00 19.14 4.14	3.33 2.78 3.82	11.04	<0.001
	*N*	*NO*	*YES*	*% (95% CI)*
*Response* ^b^	37	15	22	59.46 (43.40, 74.25)
*Remission* ^c^	37	17	20	54.05 (38.13, 69.43)

a Using last observation carried forward strategy for missing data.

b Response defined as a 50% decrease in the HRSD-17 total score from baseline to endpoint.

c Remission defined as a HRSD-17 total score ≤7 at the endpoint.

HRSD-17, 17-item Hamilton Rating Scale for Depression; GAD-7, Generalized Anxiety Disorder Scales 7-item; QOL-6, Quality of Life 6-item.

## Discussion

This proof of concept study has shown that RLIPC was safe and well tolerated in patients with MDD. Therefore, large, randomized, placebo or active-controlled, monotherapy or adjunctive to an antidepressant studies are needed to explore its efficacy in different stages of the treatment of MDD. Since this procedure can be done at home with minimal side effect, this approach especially appeals to those who are sensitive to medications and do not have cardiovascular diseases and bleeding-prone diathesis.

The long-term safety of RLIPC was demonstrated in patients with severe CAS (16) and patients with ICAS (21). RLIPC had no significant effect on the heart rate, oxygenation index, and mean flow velocity in patients with both ICAS and healthy volunteers (15). Only one patient discontinued the present study because of numbness of limbs and petechiae of skin, which was lower than that reported in CAS patients (9.5%) (16). The seven patients prematurely discontinued the study because they were discharged home. These patients might not prematurely discontinue the study if they stayed in the hospital. Without considering these seven patients, about 16% of patients prematurely discontinued who did not undergo the procedure per the treatment protocol, which was higher than those in CAS patients (RLIPC group 1.6% and sham RLIPC group 4.8%) (16). The discrepancy could be interpreted as MDD patients had poorer treatment compliance to RLIPC than CAS patients. In routine outpatient settings, up to one in five individuals may not be adherent to the prescribed antidepressants (22, 23). About one third of patients with depression could drop out from short-term neurostimulation treatment such as transcranial direct current stimulation (tDCS) and repetitive transcranial magnetic stimulation (rTMS) (24). Since a maximum of 20% of missing sessions was considered reasonable for adherence, our study suggests that RLIPC in MDD can be a reasonable method for treatment adherence.

In terms of efficacy, antidepressant combined with RLIPC significantly reduced depressive and anxious symptoms and improved the quality of life in patients with MDD as early as at the end of second week after an add-on therapy of RLIPC. By that time, all enrolled patients had taken an antidepressant(s) for 4 weeks or more. Thus, it is difficult to clarify the possible add-on effect and onset time of RLIPC. However, a remission rate of close to 60% was twice as high as that of after an antidepressant monotherapy in effectiveness clinical trials (25, 26). A possible explanation was that the inpatients were enrolled in this study that might have a higher remission rate than outpatients with MDD. Clearly, it is necessary to investigate whether the earlier response and a higher remission rate in this study were due to a true effect of adjunctive treatment of RLIPC to an antidepressant(s) or an effect of different study settings. Although the placebo effect of such a device used in ischemia patients was not significant compared the sham group with control group (16), which is necessary to be further verified in different population.

## Limitation

This study was limited due to open label, small sample size, only inpatients, and lack of clear definition of treatment stages. The additional limitation include: 1) it lack of enough information of drug use, thus may difficult to stratify the effect of drug or the add-on effect of RLIPC; 2) It also lack of data of placebo effect of such a device in the patients with MDD. However, this study achieved its goal by demonstrating the safety and tolerability, and possible efficacy in MDD with RLIPC.

## Conclusions

RLIPC, a simple and noninvasive therapy, appeared to be safe and well-tolerated in patients with MDD. Large studies including inpatients and outpatients with clearly defined treatment stages are warranted to test its efficacy and safety as monotherapy or adjunctive therapy in the treatment of MDD. Such an endeavor may expand our current available tools to manage MDD.

## Data Availability Statement

The datasets generated for this study are available on request to the corresponding authors.

## Ethics Statement

The studies involving human participants were reviewed and approved by Institutional Review Board of Hongkou District Mental Health Center (Shanghai, China). The patients/participants provided their written informed consent to participate in this study.

## Author Contributions

ZW and XL contributed to design, analysis and interpretation of data, and drafting the manuscript. KG and MR contributed to conception, design, and revising the manuscript. NL, JL, BY, JS, LH, and YF contributed the subjects’ enrollment and the clinical assessments. XJ is one of the inventors of the electric autocontrol device that has been patented in China (ZL 2007 1 017670L 0) who supplied freely all devices in this study. All authors contributed to the article and approved the submitted version.

## Conflict of Interest

XJ is one of the inventors of the electric autocontrol device that has been patented in China (ZL 2007 1 017670L 0) who supplied freely all devices in this study.

The remaining authors declare that the research was conducted in the absence of any commercial or financial relationships that could be construed as a potential conflict of interest.
